# Biomimicry and Bioinspiration as Tools for the Design of Innovative Materials and Systems

**DOI:** 10.1155/2018/6103537

**Published:** 2018-06-06

**Authors:** Saurabh Das, B. Kollbe Ahn, Nadine R. Martinez-Rodriguez

**Affiliations:** ^1^Chemical Engineering, University of California, Santa Barbara, CA 93106, USA; ^2^Materials Research Laboratory, Materials Research Science and Engineering Center, University of California, Santa Barbara, CA 93106, USA; ^3^Marine Science Institute, University of California, Santa Barbara, CA 93106, USA; ^4^Chemistry, University of Central Florida, Orlando, FL 94305, USA; ^5^Stanford School of Medicine, Stanford, CA 94305, USA

Biological systems found in nature have inspired the study and design of engineering systems and modern technology. For instance, some species of geckos have a unique ability to quickly adhere to smooth vertical surfaces without the use of liquids or surface tension. The biomimetic gecko adhesive mechanism has influenced engineers to design and build adhesive platforms aimed at achieving robust and efficiently scaled adhesion for climbing on inverted surfaces with extreme topographical features. Similarly, sandcastle worms use a unique complex coacervate to glue together sand granules on the ocean floor whereas marine mussel adhesion is less specific and certainly more opportunistic than typical ligand-receptor interactions in protein adhesion. Adhesion in such marine organism is noteworthy due to its ability to overcome moisture and its ability to realize strong and reliable adhesion under water that most fabricated adhesives are lacking. Nature has developed surprisingly varied and, at times, rather ingenious lubrication strategies for controlling and regulating the interaction forces, friction, and wear at sheared interfaces; for example, the superlubricity and wear protection properties conferred by the complex synergy between the various proteins, polysaccharides, and lipids in the synovial fluid between articular joints in animals are rather startling. In this special issue, authors have discussed biomimicry techniques and ideas to engineer materials for imprinting technology, antibacterial surfaces, drag mitigating interfaces, impact-resistant materials, and load bearing composites. Mathematical models have been used to explain the physics of the superior properties of the biomimetic materials. The practical use of the published research has been demonstrated by prototypes that mimic biological organisms.

*Saurabh Das* *B. Kollbe Ahn* *Nadine R. Martinez-Rodriguez*

